# Medical Convention of Ohio

**Published:** 1842-06

**Authors:** 


### THE WESTERN JOURNAL.

Vol. V.—No. VI.

LOUISVILLE, JUNE 1, 1842.

### MEDICAL CONVENTION OF OHIO.

The fifth meeting of this body began in Cincinnati, May 16th, at 10 o'clock, in the Hall of the Medical College. About 70 members attended, more than half of whom belonged to the city. Dr. Thompson, of Columbus, was elected President. Dr. Boerstler of Lancaster, late President, opened the sitting, and after his successor took the Chair, delivered an interesting discourse on the ætiological and therapeutic influences of heat and cold. A full narrative of the proceedings of this body, will soon come before the profession in a printed form. We shall, however, select a few matters from our own notes.

A resolution was passed inviting physicians from neighboring States to take seats as honorary members, with the privilege of participating in all the scientific exercises-

It was also resolved that no physician who is the author or vender of a patent medicine or nostrum, should have a seat in the Convention; which sound and salutary restriction we were sorry to see neglected in practice. If our conventions cannot be made up exclusively of highly scientific men, they should at least include only those who technically belong to that *caste*, and have a degree of professional self-respect that will defend them against palpable empiricism.

While on this subject we may state, that a resolution was passed, censuring the practice now becoming so prevalent with clergymen, of recommending nostrums; and, also, that a memorial to Congress was adopted, praying that the law authorizing patents for medicines may be repealed.

We were happy to observe that a committee was raised to investigate the remote causes and prevention of the *chronic laryngitis* of public speakers, generally but improperly called bronchitis.

Among other resolutions, we may mention one condemning the use of iron chains and manacles, in the treatment, or rather confinement, of insane persons; which it seems is still practised in some of the asylums of the West.

We must not omit to mention, that a resolution, declaring it a professional duty for physicians to promote, both by precept and example, the present glorious reform in the abuse of intoxicating drinks, was unanimously adopted; and we are happy to be able to state, that in most of the hospitalities extended to the members, during the whole session, wine and other intoxicating drinks were not presented.

Dr. Thompson exhibited to the Convention some surgical instruments, which he conceived to be improvements on those previously used. One was a catlin with a concave back, which will apply itself to the bone; another a cataract knife, with the blade set at an angle of a few degrees with the handle; a third was a tourniquet entirely metallic, and designed to avoid venous compression.

Dr. Mussey offered to the notice of the Convention some specimens of fracture of the neck of the thigh bone within the capsular ligament, in which a cure had taken place by ossific union.

A resolution declaring it un-professional to attend families by the year for a stipulated sum, was voted down.

It was by resolution desired, that all the members of the next Convention report what they can from their own observations on the symptoms, pathological anatomy, and treatment of Bilious Fever.

The following request was ordered to be entered on the journal of the Convention:

Snake-bites. Dr. Drake respectfully requests the members of the Convention and the readers of its proceedings, to favor him with such facts concerning the bites of our venomous snakes, as may have fallen under their own observation, or that of persons qualified to observe. He is especially desirous of learning whether the symptoms produced by the bite of the rattle-snake, the copper head, and the prairie rattle-snake, are the same; whether there is an annual recurrence of any of these symptoms; and to what extent confidence should be placed in the efficacy of those native plants which have been recommended as antidotes.

We have already spoken of the address of the late President, Dr. Boerstler, on leaving the chair; and we will now enumerate the reports and other papers which were read in the Convention. Two of these were by Dr. Harrison, one on the modus operandi of medicines, the other on fever; Dr. Mussey read a lecture on syphilis; Dr. Worcester on the diagnosis of diseases of the heart; Dr. Dawson a report on trembles and milk-sickness; and Dr. Boerstler, from Dr. Bigelow, a botanical description of some plants to which the former of those diseases has been ascribed; Dr. Thompson made a report in favor of mesmerism, which was accompanied by some mesmeric experiments; and during the discussion which followed, Dr. Drake introduced an account of some experiments recently performed by himself, which will be found in our next number; Dr. Barbee read a paper on the influence of malaria, in giving an intermittent character to certain diseases which do not depend on it; Dr. Wright a paper on certain prejudices against the medical profession; Dr. Hempstead a paper on the climate, topography, and diseases of the lower part of the valley of the Scioto; Dr. Atlee an essay on organic life, and Dr. Gross an experimental paper on wounds of the intestines.

All these papers, except the last, were referred to a committee of five members, to select from them such as they may deem proper for publication. We shall not, therefore, say any thing of them at this time, but await the apppearance of the publication, when we shall notice them according to our views of their originality and value. Dr. Gross' paper, which, by his own request, will not be published in the proceedings of the Convention, presented the general results of upwards of 40 experiments on animals, instituted with a view to ascertain the best kind of suture applicable in wounds of the intestines. Since the reading of the paper, we learn that the author has resumed his researches, and that we may shortly expect a full account of them for the pages of the Journal.

We congratulate our brethren of the western part of Ohio on the success of this, the first session of the convention held in the city of Cincinnati. It continued from Monday morning till Friday evening, two days longer than any of the other conventions; and the greater part of four days was devoted to the reading and discussion of papers. We cannot say that all these papers and discussions were of equal interest, but we are bound to admit, that many of them were both instructive and exciting. They would, however, have been more interesting, had a greater number of members participated in the debate.

To report on a meeting of 70 Doctors, without speaking of harmony, would be a great omission. At one time, the modest and orderly maid seemed in danger of being trampled under foot; but all hearts came to her rescue, and the last two days of the Convention presented as much courtesy and kindness of feeling, as we ever witnessed, in any assembly, even of our *clerical* brethren. It was the serenity and brightness which follow the *clearing up* shower, in whose *falling drops* the bow of better promise was painted in brilliant hues.

With unanimity, and much *good taste*, the Convention adjourned to meet at Lancaster next year, in *strawberry time*.

